# Biochemical Abnormalities Associated With Sudden Infant Death Syndrome: A Case Report

**DOI:** 10.7759/cureus.55292

**Published:** 2024-02-29

**Authors:** Roshani S Ganjare, Anjali A Vagga, Archana Dhok, Ashish Anjankar, Roshan K Jha, Pratiksha S Batulwar

**Affiliations:** 1 Biochemistry, Jawaharlal Nehru Medical College, Datta Meghe Institute of Higher Education & Research, Wardha, IND

**Keywords:** outcome, treatment, diagnosis, investigation, case report, risk factors, unexplained death, infant mortality, sudden infant death syndrome sids

## Abstract

Sudden infant death is a complex event characterized by biochemical features that are difficult to understand in general settings. Herein, we present a case report of a three-month-old infant who succumbed to sudden infant death syndrome (SIDS), focusing on the biochemical abnormalities identified through post-mortem analysis. The infant, previously healthy and meeting developmental milestones, was found lifeless in the crib during sleep. An autopsy revealed no anatomical abnormalities or signs of external trauma, consistent with SIDS diagnosis. Biochemical analysis of SIDS continued after post-mortem samples revealed dysregulation in neurotransmitter pathways, particularly serotonin, within the brain stem. These findings suggest a potential disruption in serotonin signaling, which may contribute to the vulnerability of infants to sudden death during sleep. Furthermore, metabolic profiling revealed deficiencies in enzymes involved in mitochondrial energy metabolism, particularly those related to fatty acid oxidation. These metabolic disturbances may compromise cellular function and contribute to the pathogenesis of SIDS. Environmental factors were also explored, with analysis revealing elevated levels of nicotine metabolites in post-mortem samples, suggesting maternal smoking exposure during pregnancy. Nicotine and its derivatives have known effects on neurotransmitter systems, potentially exacerbating underlying biochemical vulnerabilities in susceptible infants. This case report underscores the complex interplay of biochemical factors in the pathogenesis of SIDS and highlights the importance of multidisciplinary approaches in unraveling its mysteries. Further research is warranted to elucidate the precise mechanisms underlying these biochemical abnormalities and to develop targeted interventions aimed at reducing the incidence of SIDS and safeguarding infant health.

## Introduction

Sudden infant death syndrome (SIDS) is a tragic and mysterious phenomenon characterized by the sudden and unexplained death of an apparently healthy infant, typically during sleep. While the exact cause of SIDS remains unknown, research suggests that it may result from a combination of factors involving the infant’s environment, development, and underlying vulnerabilities. Biochemical processes within the body may play a significant role in predisposing infants to SIDS [1]. One of the leading theories regarding the biochemical basis of SIDS involves abnormalities in neurotransmitter regulation, particularly serotonin. Serotonin is a neurotransmitter that plays a crucial role in regulating various physiological functions, including breathing, heart rate, and temperature control. Studies have shown that some infants who succumb to SIDS have abnormalities in serotonin receptors or levels in regions of the brain stem responsible for controlling vital functions [1]. These abnormalities may disrupt the infant’s ability to respond appropriately to environmental stressors, such as changes in oxygen levels during sleep, increasing the risk of sudden death. Furthermore, disturbances in metabolic processes, such as impaired energy metabolism or deficiencies in certain enzymes, have also been implicated in SIDS [2]. These metabolic abnormalities may compromise the infant’s ability to maintain homeostasis, particularly during sleep when metabolic demands are reduced. For example, deficiencies in enzymes involved in fatty acid metabolism or mitochondrial function could impair the infant’s ability to generate energy efficiently, leading to cellular dysfunction and increased vulnerability to sudden death [3].

Additionally, environmental factors such as exposure to cigarette smoke, which contains toxins that can interfere with cellular function and oxygen delivery, have been linked to an increased risk of SIDS. Smoke exposure may disrupt respiratory and cardiovascular function in susceptible infants, exacerbating underlying biochemical abnormalities and further increasing the likelihood of sudden death [4]. In summary, while the precise biochemical mechanisms underlying SIDS remain elusive, research suggests that abnormalities in neurotransmitter regulation, metabolic processes, and environmental factors may contribute to this tragic syndrome. Understanding these biochemical pathways and risk factors is essential for developing strategies to prevent SIDS and safeguard infant health and well-being [5].

## Case presentation

**Figure 1:**
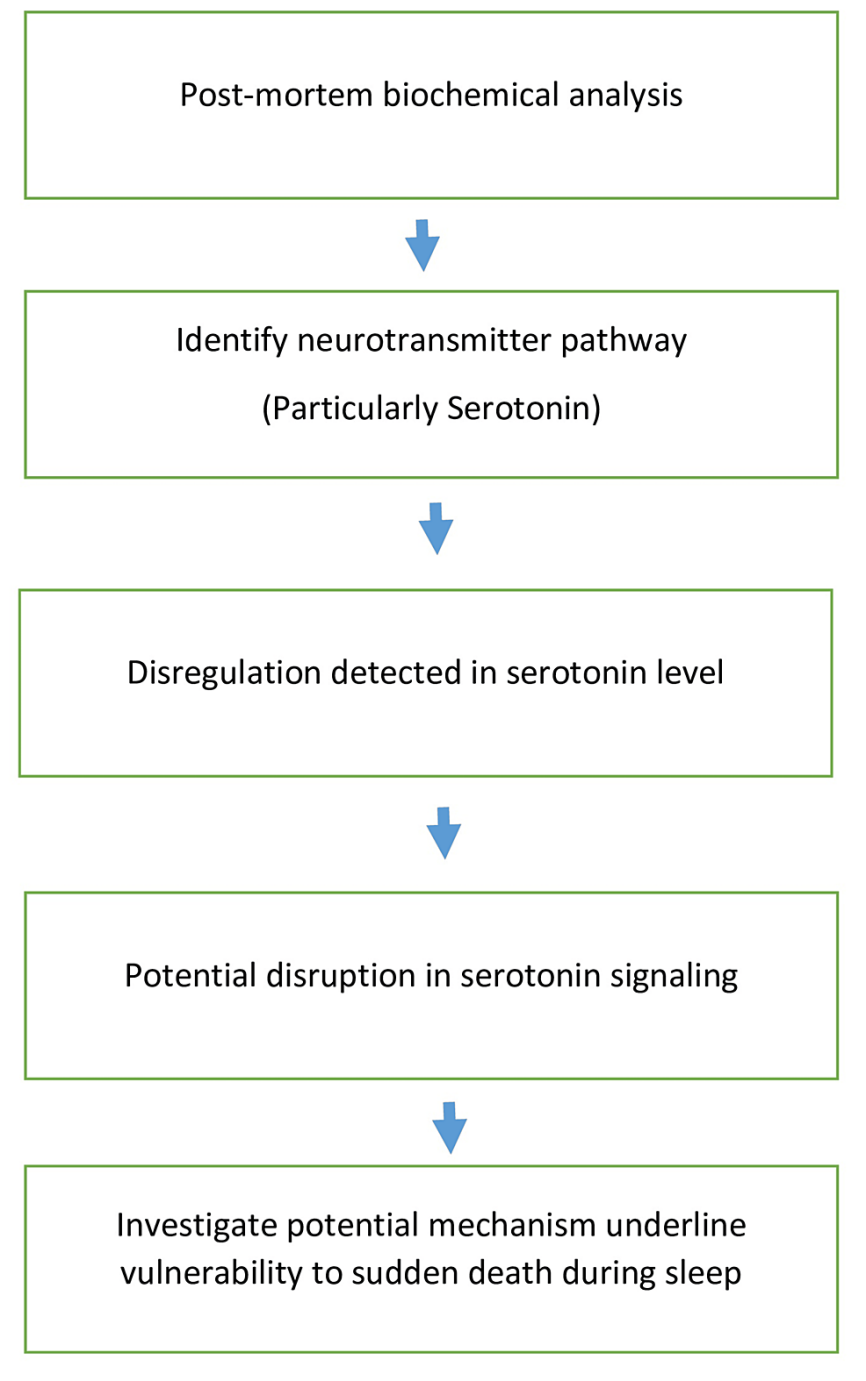
Post-mortem biochemical analysis

The infant under consideration in this case report was a three-month-old, previously healthy baby residing in the Acharya Vinoba Bhave Rural Hospital (AVBRH) Sawangi, Wardha. The parents reported that the infant was put to sleep in a standard crib on their back, adhering to recommended safe sleep practices. No significant medical history, recent illnesses, or concerns had been noted leading up to the incident. Following a routine feeding around 9 PM on the evening in question, the parents settled their infant into the crib, noting the child’s apparent good health and aspects. However, the next morning, around 6 AM, the parents discovered the infant unresponsive, prompting them to seek immediate assistance from emergency services. Upon reaching the hospital, the infant was pronounced deceased, prompting the commencement of investigations to ascertain the cause of death. The medical team conducted a comprehensive physical examination, taking into account potential external factors and evaluating the overall health status of the infant. Subsequently, an autopsy revealed no anatomical abnormalities or signs of external trauma, consistent with SIDS diagnosis. Biochemical analysis of post-mortem samples revealed dysregulation in neurotransmitter pathways, particularly serotonin, within the brain stem (Figure 1).

According to the mother, she discovered him lying on his side with a quilt covering his head, his hands tightly clenched to the quilt. His diaper was soiled with stool, and there were no signs of vomiting in the crib. She was sleeping nearby and did not notice any crying during the night. The baby was delivered at term via spontaneous vaginal delivery at the AVBRH hospital labor room. The mother was 25 years old, and the father was 29. The mother’s positive history confirmed that she had smoked during the antenatal period. She conceived shortly after four months of marriage, experiencing no complications during pregnancy and attending regular antenatal check-ups. Following delivery, the baby was healthy, weighing 2.5 kg and measuring 44 centimeters in length.

Investigation

A thorough investigation was done, which included checking the baby’s medical records and family history. An autopsy revealed no anatomical abnormalities or signs of external trauma, consistent with SIDS diagnosis. Biochemical analysis of post-mortem samples revealed dysregulation in neurotransmitter pathways, particularly serotonin, within the brain stem. Serotonin levels were checked using VITROS 5600 (Ortho Clinical Diagnostics, Raritan, New Jersey, United States), and gamma-aminobutyric acid and oxidative stress were also measured. The focus was on finding any possible reasons or irregularities that might have caused the sudden death.

Diagnosis

The diagnosis of SIDS involves the exclusion of other possible causes of death through a thorough investigation. An autopsy revealed no anatomical abnormalities or signs of external trauma, consistent with SIDS diagnosis. Biochemical analysis of post-mortem samples revealed dysregulation in neurotransmitter pathways, particularly serotonin, within the brain stem. After checking for known medical conditions and other external factors that could have caused the infant’s death, it was confirmed that the diagnosis was SIDS.

## Discussion

This case stresses the importance of the identified biochemical anomalies, specifically focusing on the disruption in neurotransmitter pathways, notably serotonin, and metabolic imbalances observed in the infant. These discoveries align with existing literature suggesting a connection between serotonin signaling abnormalities and metabolic dysfunction in the development of SIDS [1]. By furnishing concrete evidence from a particular case, this report enriches the comprehension of the biochemical underpinnings of SIDS and lends support to the notion that disruptions in these pathways may predispose infants to sudden death during sleep. Furthermore, the case explores the potential interplay between genetic predisposition and environmental factors in the occurrence of SIDS [6]. While genetic factors like serotonin receptor abnormalities and metabolic enzyme deficiencies may contribute to individual susceptibility, environmental exposures such as maternal smoking during pregnancy can exacerbate these susceptibilities. Recognizing the intricate interaction between genetic and environmental factors is vital for devising effective preventive measures and targeted interventions to reduce the incidence of SIDS [5].

Moreover, the limitations of the study include its retrospective nature and the challenge of establishing a causal relationship between the identified biochemical irregularities and SIDS [7]. While the findings offer valuable insights into potential pathophysiological mechanisms, further prospective studies involving larger cohorts are necessary to validate these findings and clarify causal relationships. Additionally, the case highlights the clinical implications of the findings by emphasizing the importance of incorporating biochemical markers into SIDS risk assessment and diagnostic protocols [1]. Biomarkers indicative of serotonin dysregulation or metabolic dysfunction could facilitate the identification of infants at heightened risk of SIDS, enabling targeted monitoring and intervention strategies to prevent sudden death [1].

**Figure 2:**
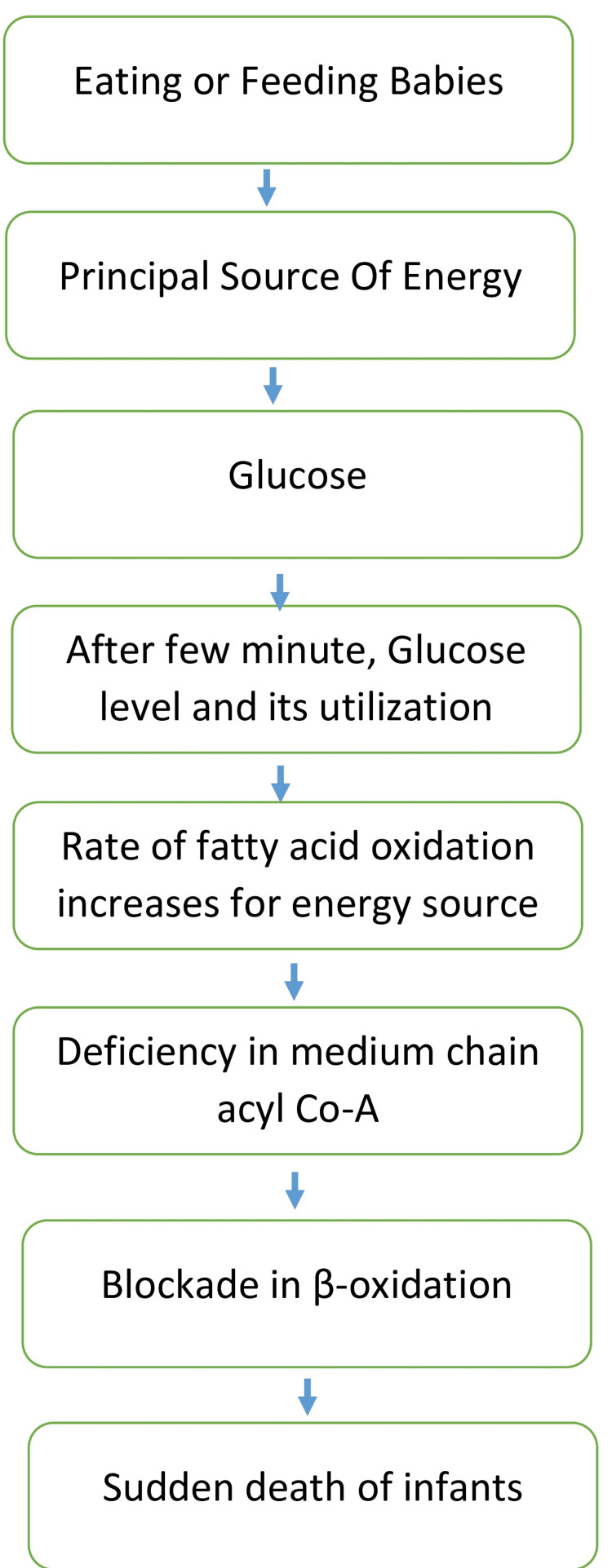
Mechanism of β-oxidation of fatty acid in SIDS SIDS, sudden infant death syndrome

Additionally, the metabolic profiling conducted in the case report targeted tissues such as the brain, heart, liver, and blood samples obtained post-mortem, seeking deviations in metabolite levels associated with key metabolic pathways like energy production, amino acid metabolism, and lipid metabolism [7]. Specifically, abnormalities could signify deficiencies in enzymes related to mitochondrial energy metabolism, particularly fatty acid oxidation. Mitochondria, responsible for adenosine triphosphate (ATP) generation, are crucial for cellular function; any dysfunction here could decrease ATP production, contributing to infant vulnerability to sudden death [8]. The mechanism of β-oxidation of fatty acid in SIDS is diagrammatically explained in Figure 2.

Furthermore, abnormal metabolite levels may indicate oxidative stress or cellular damage, which occurs when reactive oxygen species production surpasses antioxidant defenses [9]. Overall, this profiling aimed to uncover metabolic irregularities underlying SIDS, offering insights into its biochemical basis and informing future prevention strategies.

## Conclusions

The case report significantly contributed to our understanding of the biochemical basis of SIDS and underscored the importance of multidisciplinary approaches to unraveling its complexities. By elucidating the role of neurotransmitter abnormalities and metabolic disturbances, this study provided valuable insights into potential preventive strategies and laid the groundwork for future research aimed at reducing the burden of SIDS on affected families and communities.
